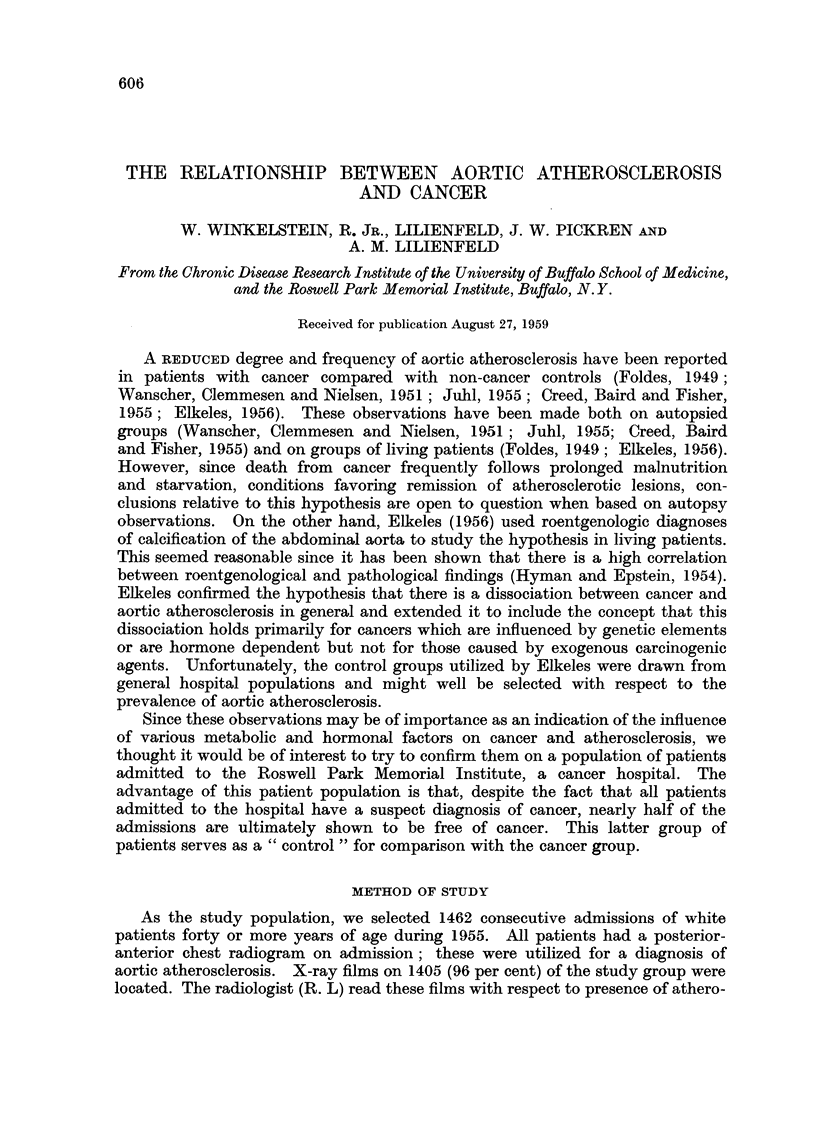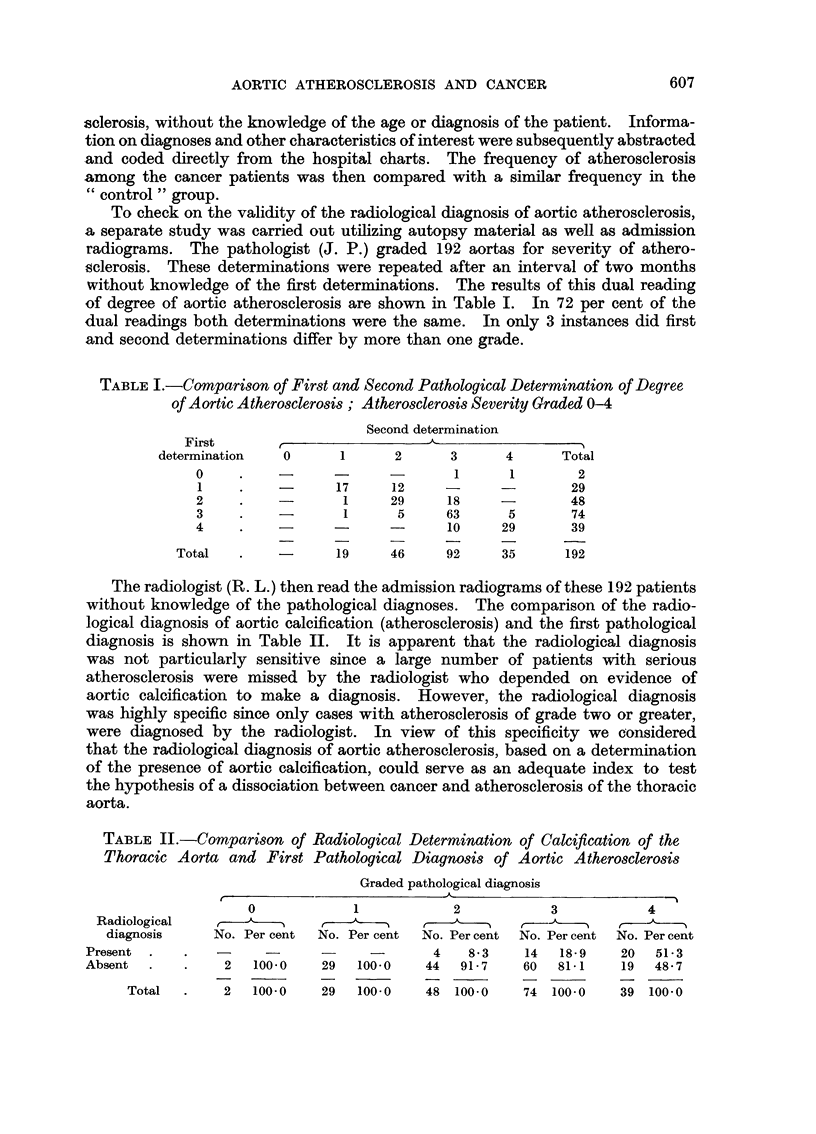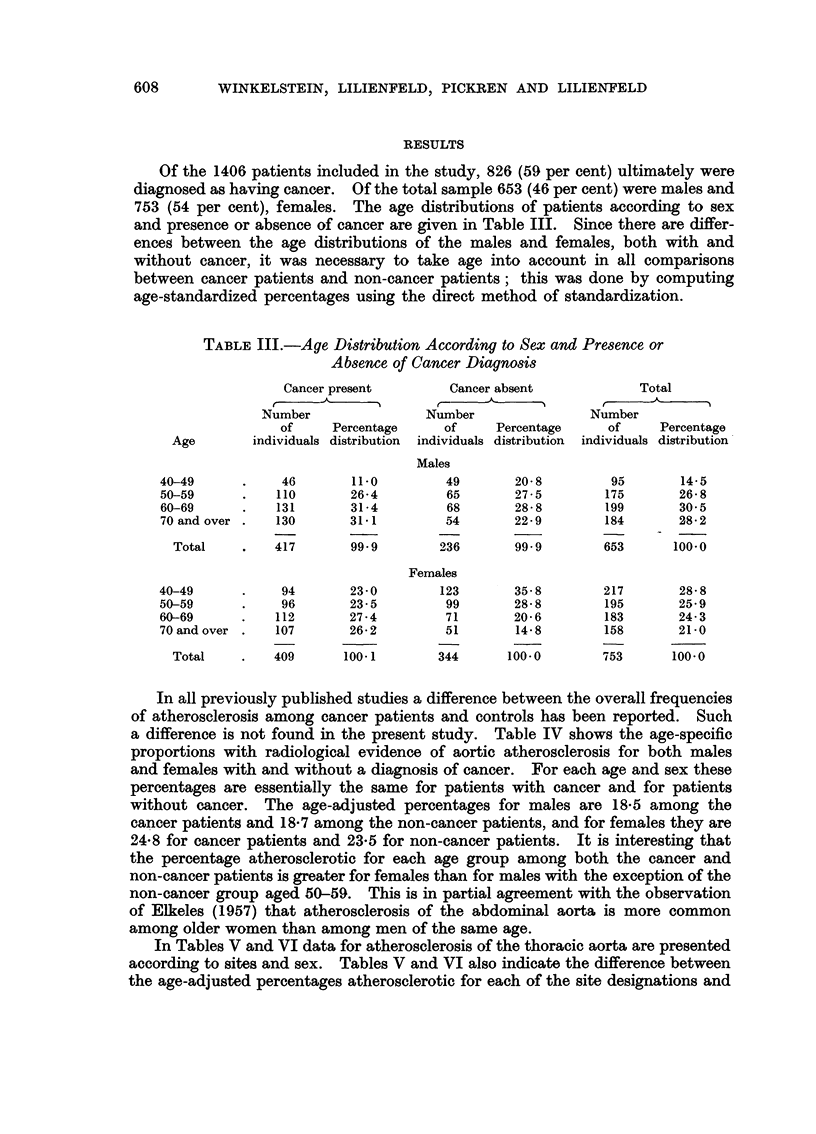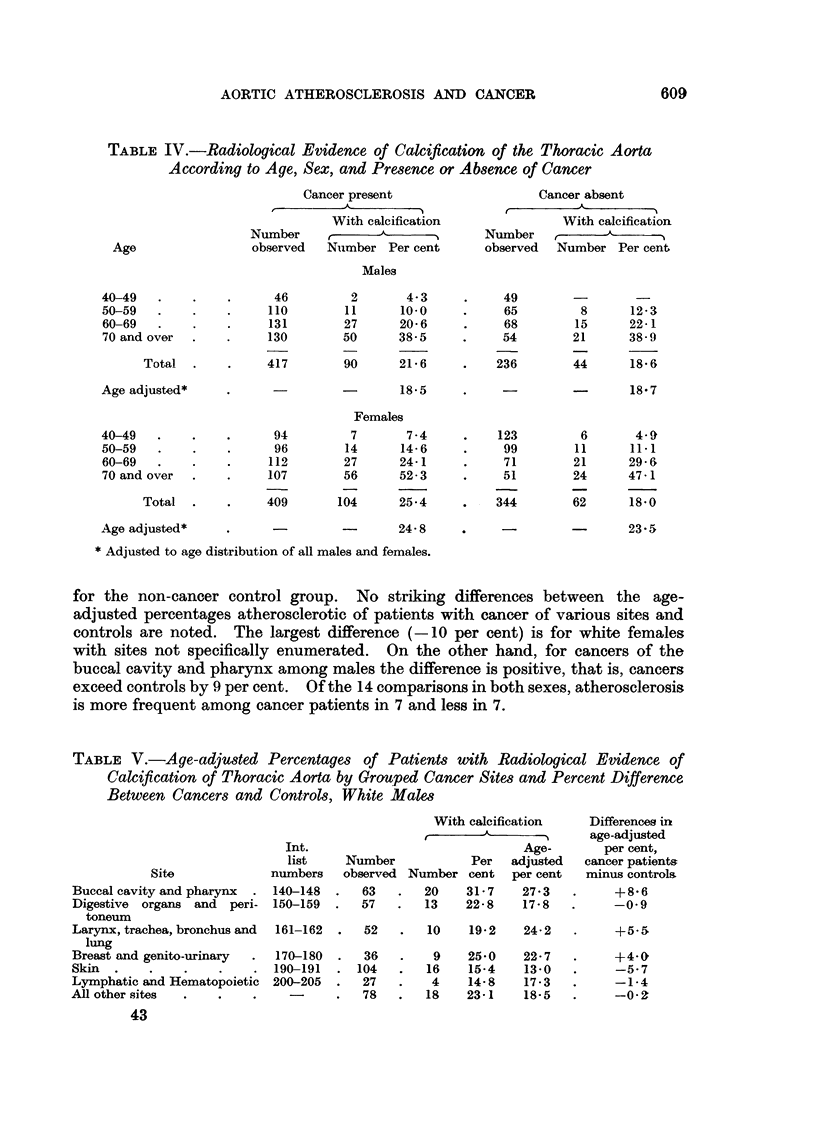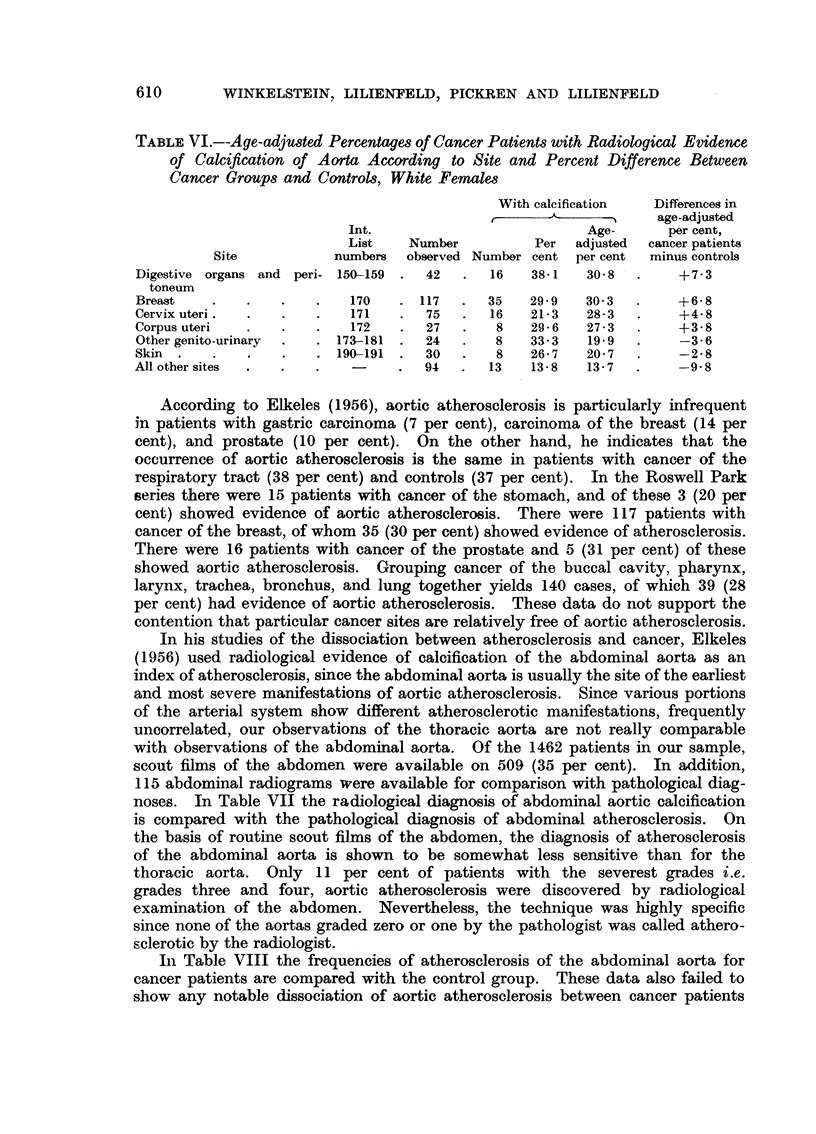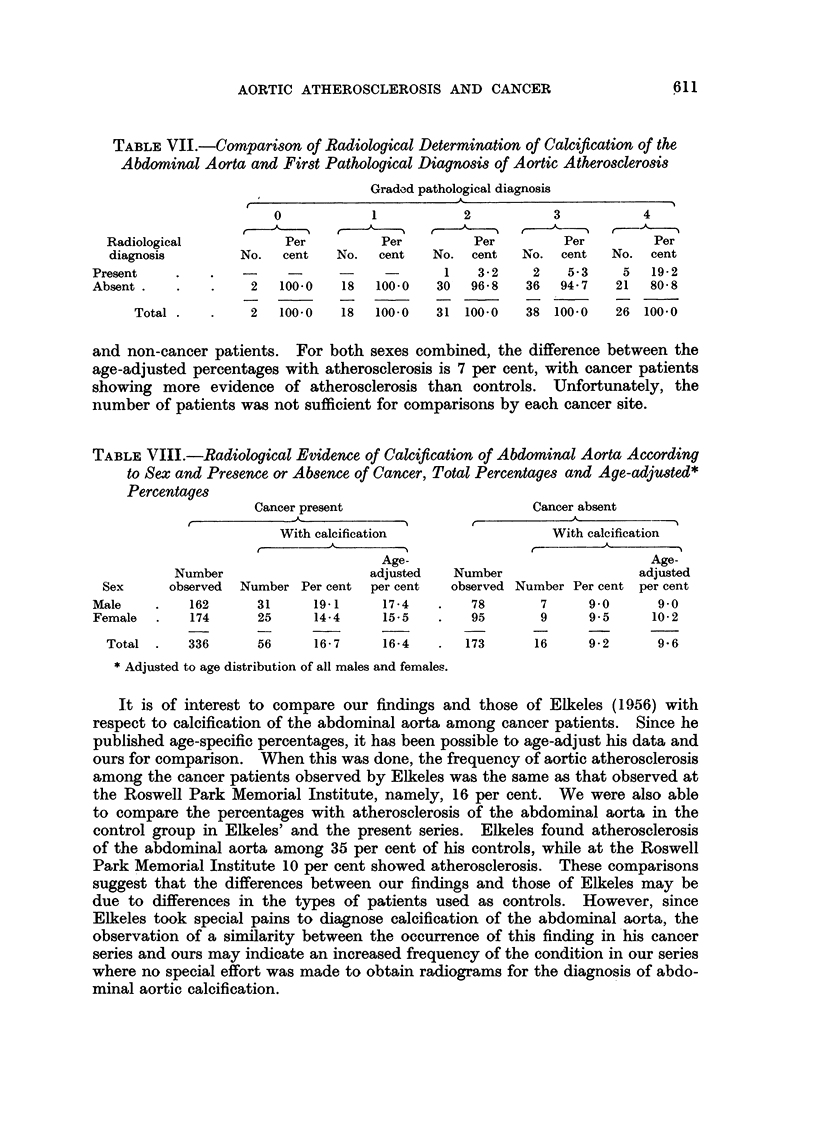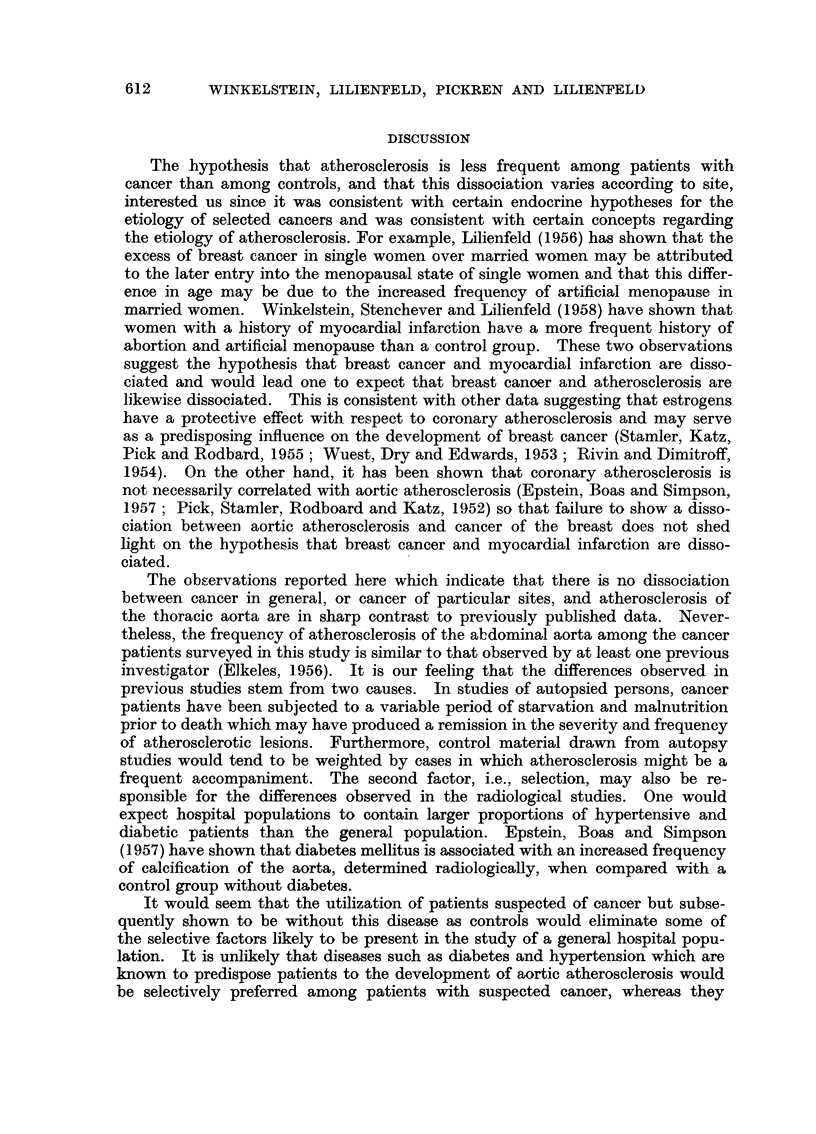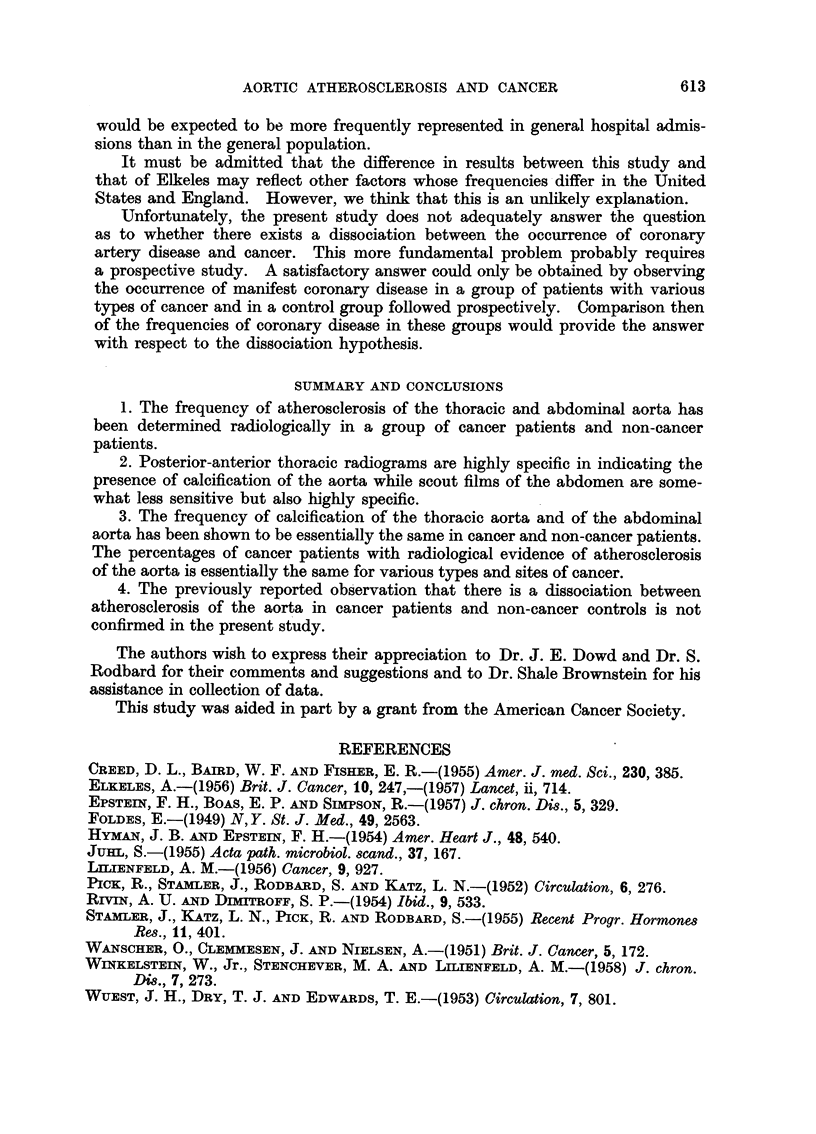# The Relationship Between Aortic Atherosclerosis and Cancer

**DOI:** 10.1038/bjc.1959.65

**Published:** 1959-12

**Authors:** W. Winkelstein, R. Lilienfeld, J. W. Pickren, A. M. Lilienfeld


### THE RELATIONSHIP BETWEEN AORTIC ATHEROSCLEROSIS AND CANCER

W. WINKELSTEIN, R. JR., LILIENFELD, J. W. PICKREN AND A. M. LILIENFELD

*From the Chronic Disease Research Institute of the University of Buffalo School of Medicine, and the Roswell Park Memorial Institute, Buffalo, N.Y.*

Received for publication August 27, 1959

A REDUCED degree and frequency of aortic atherosclerosis have been reported in patients with cancer compared with non-cancer controls (Foldes, 1949; Wanscher, Clemmesen and Nielsen, 1951; Juhl, 1955; Creed, Baird and Fisher, 1955; Elkeles, 1956). These observations have been made both on autopsied groups (Wanscher, Clemmesen and Nielsen, 1951; Juhl, 1955; Creed, Baird and Fisher, 1955) and on groups of living patients (Foldes, 1949; Elkeles, 1956). However, since death from cancer frequently follows prolonged malnutrition and starvation, conditions favoring remission of atherosclerotic lesions, conclusions relative to this hypothesis are open to question when based on autopsy observations. On the other hand, Elkeles (1956) used roentgenologic diagnoses of calcification of the abdominal aorta to study the hypothesis in living patients. This seemed reasonable since it has been shown that there is a high correlation between roentgenological and pathological findings (Hyman and Epstein, 1954). Elkeles confirmed the hypothesis that there is a dissociation between cancer and aortic atherosclerosis in general and extended it to include the concept that this dissociation holds primarily for cancers which are influenced by genetic elements or are hormone dependent but not for those caused by exogenous carcinogenic agents. Unfortunately, the control groups utilized by Elkeles were drawn from general hospital populations and might well be selected with respect to the prevalence of aortic atherosclerosis.

Since these observations may be of importance as an indication of the influence of various metabolic and hormonal factors on cancer and atherosclerosis, we thought it would be of interest to try to confirm them on a population of patients admitted to the Roswell Park Memorial Institute, a cancer hospital. The advantage of this patient population is that, despite the fact that all patients admitted to the hospital have a suspect diagnosis of cancer, nearly half of the admissions are ultimately shown to be free of cancer. This latter group of patients serves as a " control " for comparison with the cancer group.

### METHOD OF STUDY

As the study population, we selected 1462 consecutive admissions of white patients forty or more years of age during 1955. All patients had a posterior-anterior chest radiogram on admission; these were utilized for a diagnosis of aortic atherosclerosis. X-ray films on 1405 (96 per cent) of the study group were located. The radiologist (R. L) read these films with respect to presence of atherosclerosis, without the knowledge of the age or diagnosis of the patient. Information on diagnoses and other characteristics of interest were subsequently abstracted and coded directly from the hospital charts. The frequency of atherosclerosis among the cancer patients was then compared with a similar frequency in the " control " group.

To check on the validity of the radiological diagnosis of aortic atherosclerosis, a separate study was carried out utilizing autopsy material as well as admission radiograms. The pathologist (J. P.) graded 192 aortas for severity of atherosclerosis. These determinations were repeated after an interval of two months without knowledge of the first determinations. The results of this dual reading of degree of aortic atherosclerosis are shown in Table I. In 72 per cent of the dual readings both determinations were the same. In only 3 instances did first and second determinations differ by more than one grade.

TABLE I.—*Comparison of First and Second Pathological Determination of Degree of Aortic Atherosclerosis ; Atherosclerosis Severity Graded* 0–4

| First determination | Second determination 0 | 1 | 2 | 3 | 4 | Total |
|---|---|---|---|---|---|---|
| 0 | — | — | — | 1 | 1 | 2 |
| 1 | — | 17 | 12 | — | — | 29 |
| 2 | — | 1 | 29 | 18 | — | 48 |
| 3 | — | 1 | 5 | 63 | 5 | 74 |
| 4 | — | — | — | 10 | 29 | 39 |
| Total | — | 19 | 46 | 92 | 35 | 192 |

The radiologist (R. L.) then read the admission radiograms of these 192 patients without knowledge of the pathological diagnoses. The comparison of the radiological diagnosis of aortic calcification (atherosclerosis) and the first pathological diagnosis is shown in Table II. It is apparent that the radiological diagnosis was not particularly sensitive since a large number of patients with serious atherosclerosis were missed by the radiologist who depended on evidence of aortic calcification to make a diagnosis. However, the radiological diagnosis was highly specific since only cases with atherosclerosis of grade two or greater, were diagnosed by the radiologist. In view of this specificity we considered that the radiological diagnosis of aortic atherosclerosis, based on a determination of the presence of aortic calcification, could serve as an adequate index to test the hypothesis of a dissociation between cancer and atherosclerosis of the thoracic aorta.

TABLE II.—*Comparison of Radiological Determination of Calcification of the Thoracic Aorta and First Pathological Diagnosis of Aortic Atherosclerosis*

| Radiological diagnosis | Graded pathological diagnosis 0 | | 1 | | 2 | | 3 | | 4 | |
|---|---|---|---|---|---|---|---|---|---|---|
| | No. | Per cent | No. | Per cent | No. | Per cent | No. | Per cent | No. | Per cent |
| Present | — | — | — | — | 4 | 8·3 | 14 | 18·9 | 20 | 51·3 |
| Absent | 2 | 100·0 | 29 | 100·0 | 44 | 91·7 | 60 | 81·1 | 19 | 48·7 |
| Total | 2 | 100·0 | 29 | 100·0 | 48 | 100·0 | 74 | 100·0 | 39 | 100·0 |

### RESULTS

Of the 1406 patients included in the study, 826 (59 per cent) ultimately were diagnosed as having cancer. Of the total sample 653 (46 per cent) were males and 753 (54 per cent), females. The age distributions of patients according to sex and presence or absence of cancer are given in Table III. Since there are differences between the age distributions of the males and females, both with and without cancer, it was necessary to take age into account in all comparisons between cancer patients and non-cancer patients ; this was done by computing age-standardized percentages using the direct method of standardization.

TABLE III.—*Age Distribution According to Sex and Presence or Absence of Cancer Diagnosis*

| | Cancer present | | Cancer absent | | Total | |
|---|---|---|---|---|---|---|
| Age | Number of individuals | Percentage distribution | Number of individuals | Percentage distribution | Number of individuals | Percentage distribution |
| | | | Males | | | |
| 40–49 . | 46 | 11·0 | 49 | 20·8 | 95 | 14·5 |
| 50–59 . | 110 | 26·4 | 65 | 27·5 | 175 | 26·8 |
| 60–69 . | 131 | 31·4 | 68 | 28·8 | 199 | 30·5 |
| 70 and over . | 130 | 31·1 | 54 | 22·9 | 184 | 28·2 |
| Total . | 417 | 99·9 | 236 | 99·9 | 653 | 100·0 |
| | | | Females | | | |
| 40–49 . | 94 | 23·0 | 123 | 35·8 | 217 | 28·8 |
| 50–59 . | 96 | 23·5 | 99 | 28·8 | 195 | 25·9 |
| 60–69 . | 112 | 27·4 | 71 | 20·6 | 183 | 24·3 |
| 70 and over . | 107 | 26·2 | 51 | 14·8 | 158 | 21·0 |
| Total . | 409 | 100·1 | 344 | 100·0 | 753 | 100·0 |

In all previously published studies a difference between the overall frequencies of atherosclerosis among cancer patients and controls has been reported. Such a difference is not found in the present study. Table IV shows the age-specific proportions with radiological evidence of aortic atherosclerosis for both males and females with and without a diagnosis of cancer. For each age and sex these percentages are essentially the same for patients with cancer and for patients without cancer. The age-adjusted percentages for males are 18·5 among the cancer patients and 18·7 among the non-cancer patients, and for females they are 24·8 for cancer patients and 23·5 for non-cancer patients. It is interesting that the percentage atherosclerotic for each age group among both the cancer and non-cancer patients is greater for females than for males with the exception of the non-cancer group aged 50–59. This is in partial agreement with the observation of Elkeles (1957) that atherosclerosis of the abdominal aorta is more common among older women than among men of the same age.

In Tables V and VI data for atherosclerosis of the thoracic aorta are presented according to sites and sex. Tables V and VI also indicate the difference between the age-adjusted percentages atherosclerotic for each of the site designations and

TABLE IV.—*Radiological Evidence of Calcification of the Thoracic Aorta According to Age, Sex, and Presence or Absence of Cancer*

| Age | Cancer present: Number observed | Cancer present: With calcification: Number | Cancer present: With calcification: Per cent | Cancer absent: Number observed | Cancer absent: With calcification: Number | Cancer absent: With calcification: Per cent |
|---|---|---|---|---|---|---|
| | | Males | | | | |
| 40–49 | 46 | 2 | 4·3 | 49 | — | — |
| 50–59 | 110 | 11 | 10·0 | 65 | 8 | 12·3 |
| 60–69 | 131 | 27 | 20·6 | 68 | 15 | 22·1 |
| 70 and over | 130 | 50 | 38·5 | 54 | 21 | 38·9 |
| Total | 417 | 90 | 21·6 | 236 | 44 | 18·6 |
| Age adjusted* | — | — | 18·5 | — | — | 18·7 |
| | | Females | | | | |
| 40–49 | 94 | 7 | 7·4 | 123 | 6 | 4·9 |
| 50–59 | 96 | 14 | 14·6 | 99 | 11 | 11·1 |
| 60–69 | 112 | 27 | 24·1 | 71 | 21 | 29·6 |
| 70 and over | 107 | 56 | 52·3 | 51 | 24 | 47·1 |
| Total | 409 | 104 | 25·4 | 344 | 62 | 18·0 |
| Age adjusted* | — | — | 24·8 | — | — | 23·5 |

* Adjusted to age distribution of all males and females.

for the non-cancer control group. No striking differences between the age-adjusted percentages atherosclerotic of patients with cancer of various sites and controls are noted. The largest difference (—10 per cent) is for white females with sites not specifically enumerated. On the other hand, for cancers of the buccal cavity and pharynx among males the difference is positive, that is, cancers exceed controls by 9 per cent. Of the 14 comparisons in both sexes, atherosclerosis is more frequent among cancer patients in 7 and less in 7.

TABLE V.—*Age-adjusted Percentages of Patients with Radiological Evidence of Calcification of Thoracic Aorta by Grouped Cancer Sites and Percent Difference Between Cancers and Controls, White Males*

| Site | Int. list numbers | Number observed | With calcification: Number | With calcification: Per cent | With calcification: Age-adjusted per cent | Differences in age-adjusted per cent, cancer patients minus controls |
|---|---|---|---|---|---|---|
| Buccal cavity and pharynx | 140–148 | 63 | 20 | 31·7 | 27·3 | +8·6 |
| Digestive organs and peritoneum | 150–159 | 57 | 13 | 22·8 | 17·8 | −0·9 |
| Larynx, trachea, bronchus and lung | 161–162 | 52 | 10 | 19·2 | 24·2 | +5·5 |
| Breast and genito-urinary | 170–180 | 36 | 9 | 25·0 | 22·7 | +4·0 |
| Skin | 190–191 | 104 | 16 | 15·4 | 13·0 | −5·7 |
| Lymphatic and Hematopoietic | 200–205 | 27 | 4 | 14·8 | 17·3 | −1·4 |
| All other sites | — | 78 | 18 | 23·1 | 18·5 | −0·2 |

TABLE VI.—*Age-adjusted Percentages of Cancer Patients with Radiological Evidence of Calcification of Aorta According to Site and Percent Difference Between Cancer Groups and Controls, White Females*

| Site | Int. List numbers | Number observed | With calcification: Number | With calcification: Per cent | With calcification: Age-adjusted per cent | Differences in age-adjusted per cent, cancer patients minus controls |
|---|---|---|---|---|---|---|
| Digestive organs and peritoneum | 150–159 | 42 | 16 | 38·1 | 30·8 | +7·3 |
| Breast | 170 | 117 | 35 | 29·9 | 30·3 | +6·8 |
| Cervix uteri | 171 | 75 | 16 | 21·3 | 28·3 | +4·8 |
| Corpus uteri | 172 | 27 | 8 | 29·6 | 27·3 | +3·8 |
| Other genito-urinary | 173–181 | 24 | 8 | 33·3 | 19·9 | −3·6 |
| Skin | 190–191 | 30 | 8 | 26·7 | 20·7 | −2·8 |
| All other sites | — | 94 | 13 | 13·8 | 13·7 | −9·8 |

According to Elkeles (1956), aortic atherosclerosis is particularly infrequent in patients with gastric carcinoma (7 per cent), carcinoma of the breast (14 per cent), and prostate (10 per cent). On the other hand, he indicates that the occurrence of aortic atherosclerosis is the same in patients with cancer of the respiratory tract (38 per cent) and controls (37 per cent). In the Roswell Park series there were 15 patients with cancer of the stomach, and of these 3 (20 per cent) showed evidence of aortic atherosclerosis. There were 117 patients with cancer of the breast, of whom 35 (30 per cent) showed evidence of atherosclerosis. There were 16 patients with cancer of the prostate and 5 (31 per cent) of these showed aortic atherosclerosis. Grouping cancer of the buccal cavity, pharynx, larynx, trachea, bronchus, and lung together yields 140 cases, of which 39 (28 per cent) had evidence of aortic atherosclerosis. These data do not support the contention that particular cancer sites are relatively free of aortic atherosclerosis.

In his studies of the dissociation between atherosclerosis and cancer, Elkeles (1956) used radiological evidence of calcification of the abdominal aorta as an index of atherosclerosis, since the abdominal aorta is usually the site of the earliest and most severe manifestations of aortic atherosclerosis. Since various portions of the arterial system show different atherosclerotic manifestations, frequently uncorrelated, our observations of the thoracic aorta are not really comparable with observations of the abdominal aorta. Of the 1462 patients in our sample, scout films of the abdomen were available on 509 (35 per cent). In addition, 115 abdominal radiograms were available for comparison with pathological diagnoses. In Table VII the radiological diagnosis of abdominal aortic calcification is compared with the pathological diagnosis of abdominal atherosclerosis. On the basis of routine scout films of the abdomen, the diagnosis of atherosclerosis of the abdominal aorta is shown to be somewhat less sensitive than for the thoracic aorta. Only 11 per cent of patients with the severest grades *i.e.* grades three and four, aortic atherosclerosis were discovered by radiological examination of the abdomen. Nevertheless, the technique was highly specific since none of the aortas graded zero or one by the pathologist was called atherosclerotic by the radiologist.

In Table VIII the frequencies of atherosclerosis of the abdominal aorta for cancer patients are compared with the control group. These data also failed to show any notable dissociation of aortic atherosclerosis between cancer patients

TABLE VII.—*Comparison of Radiological Determination of Calcification of the Abdominal Aorta and First Pathological Diagnosis of Aortic Atherosclerosis*

| | Graded pathological diagnosis | | | | | | | | | |
|---|---|---|---|---|---|---|---|---|---|---|
| | 0 | | 1 | | 2 | | 3 | | 4 | |
| Radiological diagnosis | No. | Per cent | No. | Per cent | No. | Per cent | No. | Per cent | No. | Per cent |
| Present | — | — | — | — | 1 | 3·2 | 2 | 5·3 | 5 | 19·2 |
| Absent | 2 | 100·0 | 18 | 100·0 | 30 | 96·8 | 36 | 94·7 | 21 | 80·8 |
| Total | 2 | 100·0 | 18 | 100·0 | 31 | 100·0 | 38 | 100·0 | 26 | 100·0 |

and non-cancer patients. For both sexes combined, the difference between the age-adjusted percentages with atherosclerosis is 7 per cent, with cancer patients showing more evidence of atherosclerosis than controls. Unfortunately, the number of patients was not sufficient for comparisons by each cancer site.

TABLE VIII.—*Radiological Evidence of Calcification of Abdominal Aorta According to Sex and Presence or Absence of Cancer, Total Percentages and Age-adjusted* Percentages*

| | Cancer present | | | | Cancer absent | | | |
|---|---|---|---|---|---|---|---|---|
| | | With calcification | | | | With calcification | | |
| Sex | Number observed | Number | Per cent | Age-adjusted per cent | Number observed | Number | Per cent | Age-adjusted per cent |
| Male | 162 | 31 | 19·1 | 17·4 | 78 | 7 | 9·0 | 9·0 |
| Female | 174 | 25 | 14·4 | 15·5 | 95 | 9 | 9·5 | 10·2 |
| Total | 336 | 56 | 16·7 | 16·4 | 173 | 16 | 9·2 | 9·6 |

* Adjusted to age distribution of all males and females.

It is of interest to compare our findings and those of Elkeles (1956) with respect to calcification of the abdominal aorta among cancer patients. Since he published age-specific percentages, it has been possible to age-adjust his data and ours for comparison. When this was done, the frequency of aortic atherosclerosis among the cancer patients observed by Elkeles was the same as that observed at the Roswell Park Memorial Institute, namely, 16 per cent. We were also able to compare the percentages with atherosclerosis of the abdominal aorta in the control group in Elkeles' and the present series. Elkeles found atherosclerosis of the abdominal aorta among 35 per cent of his controls, while at the Roswell Park Memorial Institute 10 per cent showed atherosclerosis. These comparisons suggest that the differences between our findings and those of Elkeles may be due to differences in the types of patients used as controls. However, since Elkeles took special pains to diagnose calcification of the abdominal aorta, the observation of a similarity between the occurrence of this finding in his cancer series and ours may indicate an increased frequency of the condition in our series where no special effort was made to obtain radiograms for the diagnosis of abdominal aortic calcification.

### DISCUSSION

The hypothesis that atherosclerosis is less frequent among patients with cancer than among controls, and that this dissociation varies according to site, interested us since it was consistent with certain endocrine hypotheses for the etiology of selected cancers and was consistent with certain concepts regarding the etiology of atherosclerosis. For example, Lilienfeld (1956) has shown that the excess of breast cancer in single women over married women may be attributed to the later entry into the menopausal state of single women and that this difference in age may be due to the increased frequency of artificial menopause in married women. Winkelstein, Stenchever and Lilienfeld (1958) have shown that women with a history of myocardial infarction have a more frequent history of abortion and artificial menopause than a control group. These two observations suggest the hypothesis that breast cancer and myocardial infarction are dissociated and would lead one to expect that breast cancer and atherosclerosis are likewise dissociated. This is consistent with other data suggesting that estrogens have a protective effect with respect to coronary atherosclerosis and may serve as a predisposing influence on the development of breast cancer (Stamler, Katz, Pick and Rodbard, 1955 ; Wuest, Dry and Edwards, 1953 ; Rivin and Dimitroff, 1954). On the other hand, it has been shown that coronary atherosclerosis is not necessarily correlated with aortic atherosclerosis (Epstein, Boas and Simpson, 1957 ; Pick, Stamler, Rodboard and Katz, 1952) so that failure to show a dissociation between aortic atherosclerosis and cancer of the breast does not shed light on the hypothesis that breast cancer and myocardial infarction are dissociated.

The observations reported here which indicate that there is no dissociation between cancer in general, or cancer of particular sites, and atherosclerosis of the thoracic aorta are in sharp contrast to previously published data. Nevertheless, the frequency of atherosclerosis of the abdominal aorta among the cancer patients surveyed in this study is similar to that observed by at least one previous investigator (Elkeles, 1956). It is our feeling that the differences observed in previous studies stem from two causes. In studies of autopsied persons, cancer patients have been subjected to a variable period of starvation and malnutrition prior to death which may have produced a remission in the severity and frequency of atherosclerotic lesions. Furthermore, control material drawn from autopsy studies would tend to be weighted by cases in which atherosclerosis might be a frequent accompaniment. The second factor, i.e., selection, may also be responsible for the differences observed in the radiological studies. One would expect hospital populations to contain larger proportions of hypertensive and diabetic patients than the general population. Epstein, Boas and Simpson (1957) have shown that diabetes mellitus is associated with an increased frequency of calcification of the aorta, determined radiologically, when compared with a control group without diabetes.

It would seem that the utilization of patients suspected of cancer but subsequently shown to be without this disease as controls would eliminate some of the selective factors likely to be present in the study of a general hospital population. It is unlikely that diseases such as diabetes and hypertension which are known to predispose patients to the development of aortic atherosclerosis would be selectively preferred among patients with suspected cancer, whereas they would be expected to be more frequently represented in general hospital admissions than in the general population.

It must be admitted that the difference in results between this study and that of Elkeles may reflect other factors whose frequencies differ in the United States and England. However, we think that this is an unlikely explanation.

Unfortunately, the present study does not adequately answer the question as to whether there exists a dissociation between the occurrence of coronary artery disease and cancer. This more fundamental problem probably requires a prospective study. A satisfactory answer could only be obtained by observing the occurrence of manifest coronary disease in a group of patients with various types of cancer and in a control group followed prospectively. Comparison then of the frequencies of coronary disease in these groups would provide the answer with respect to the dissociation hypothesis.

### SUMMARY AND CONCLUSIONS

1. The frequency of atherosclerosis of the thoracic and abdominal aorta has been determined radiologically in a group of cancer patients and non-cancer patients.

2. Posterior-anterior thoracic radiograms are highly specific in indicating the presence of calcification of the aorta while scout films of the abdomen are somewhat less sensitive but also highly specific.

3. The frequency of calcification of the thoracic aorta and of the abdominal aorta has been shown to be essentially the same in cancer and non-cancer patients. The percentages of cancer patients with radiological evidence of atherosclerosis of the aorta is essentially the same for various types and sites of cancer.

4. The previously reported observation that there is a dissociation between atherosclerosis of the aorta in cancer patients and non-cancer controls is not confirmed in the present study.

The authors wish to express their appreciation to Dr. J. E. Dowd and Dr. S. Rodbard for their comments and suggestions and to Dr. Shale Brownstein for his assistance in collection of data.

This study was aided in part by a grant from the American Cancer Society.

### REFERENCES

Creed, D. L., Baird, W. F. and Fisher, E. R.—(1955) *Amer. J. med. Sci.*, **230**, 385.
Elkeles, A.—(1956) *Brit. J. Cancer*, **10**, 247,—(1957) *Lancet*, ii, 714.
Epstein, F. H., Boas, E. P. and Simpson, R.—(1957) *J. chron. Dis.*, **5**, 329.
Foldes, E.—(1949) *N,Y. St. J. Med.*, **49**, 2563.
Hyman, J. B. and Epstein, F. H.—(1954) *Amer. Heart J.*, **48**, 540.
Juhl, S.—(1955) *Acta path. microbiol. scand.*, **37**, 167.
Lilienfeld, A. M.—(1956) *Cancer*, **9**, 927.
Pick, R., Stamler, J., Rodbard, S. and Katz, L. N.—(1952) *Circulation*, **6**, 276.
Rivin, A. U. and Dimitroff, S. P.—(1954) *Ibid.*, **9**, 533.
Stamler, J., Katz, L. N., Pick, R. and Rodbard, S.—(1955) *Recent Progr. Hormones Res.*, **11**, 401.
Wanscher, O., Clemmesen, J. and Nielsen, A.—(1951) *Brit. J. Cancer*, **5**, 172.
Winkelstein, W., Jr., Stenchever, M. A. and Lilienfeld, A. M.—(1958) *J. chron. Dis.*, **7**, 273.
Wuest, J. H., Dry, T. J. and Edwards, T. E.—(1953) *Circulation*, **7**, 801.